# Physical activity and unplanned illness-related work absenteeism: Data from an employee wellness program

**DOI:** 10.1371/journal.pone.0176872

**Published:** 2017-05-04

**Authors:** Elena Losina, Heidi Y. Yang, Bhushan R. Deshpande, Jeffrey N. Katz, Jamie E. Collins

**Affiliations:** 1 Orthopaedic and Arthritis Center for Outcomes Research (OrACORe), Department of Orthopaedic Surgery, Brigham and Women’s Hospital, Boston, Massachusetts, United States of America; 2 Policy and Innovation eValuation in Orthopaedic Treatments (PIVOT) Center, Department of Orthopaedic Surgery, Brigham and Women’s Hospital, Boston, Massachusetts, United States of America; 3 Harvard Medical School, Boston, Massachusetts, United States of America; 4 Division of Rheumatology, Immunology and Allergy, Brigham and Women’s Hospital, Boston, Massachusetts, United States of America; 5 Department of Biostatistics, Boston University School of Public Health, Boston, Massachusetts, United States of America; 6 Departments of Epidemiology and Environmental Health, Harvard T. H. Chan School of Public Health, Boston, Massachusetts, United States of America; McMaster University, CANADA

## Abstract

**Background:**

Illness-related absenteeism is a major threat to work productivity. Our objective was to assess the relationship between physical activity and unplanned illness-related absenteeism from work.

**Methods:**

We implemented physical activity program for sedentary non-clinician employees of a tertiary medical center. Financial rewards were available for reaching accelerometer-measured ambulatory physical activity goals over a 24-week period. We categorized participants into three groups based on mean levels of physical activity: low (0–74 min/week), medium (75–149 min/week) and meeting CDC guidelines (≥150 min/week). We built a multivariable Poisson regression model to evaluate the relationship between physical activity and rates of unplanned illness-related absenteeism.

**Results:**

The sample consisted of 292 employees who participated in the program. Their mean age was 38 years (SD 11), 83% were female, and 38% were obese. Over the 24 intervention weeks, participants engaged in a mean of 90 min/week (SD 74) of physical activity and missed a mean of 14 hours of work (SD 38) due to illness. Unplanned absenteeism due to illness was associated with physical activity. As compared to the group meeting CDC guidelines, in multivariable analyses those in the medium physical activity group had a 2.4 (95% CI 1.3–4.5) fold higher rate of illness-related absenteeism and those in the lowest physical activity group had a 3.5 (95% CI 1.7–7.2) fold higher rate of illness-related absenteeism.

**Discussion:**

Less physical activity was associated with more illness-related absenteeism. Workforce-based interventions to increase physical activity may thus be a promising vehicle to reduce unplanned illness-related absenteeism.

## Introduction

Absenteeism from work related to illness is a major threat to work productivity, with an estimated 250 billion dollars lost annually in the US [1, 2]. Employers anticipate occasional illness-related absenteeism and its attendant effects, such as delayed task completion, hampered collaboration, and paying for sick leave [3–6]. Evolving evidence suggests that physical activity may be associated with work productivity and that increased physical activity can further lead to lower rates of work absenteeism [7, 8]. Employers are thus incentivized to offer workplace wellness programs to increase physical activity among their employees [9, 10].

The US Centers for Disease Control and Prevention (CDC) formally recommends that adults engage in at least 150 minutes of moderate-intensity aerobic physical activity or equivalent per week [11]. The positive effects of physical activity on quality of life and disease prevention have been widely documented in the literature [12–14]. Fewer than half of Americans reported meeting CDC physical activity guidelines in 2011–12 [15].

Many previous studies that investigated the relationship between physical activity and absenteeism did not objectively measure physical activity over a continuous and extended period of time [8]. We believed that such a relationship does exist and in our analysis sought to quantify the association between long-term, objectively-measured physical activity and illness-related absenteeism in a cohort of sedentary hospital employees undergoing a financial incentives-based physical activity improvement intervention.

## Materials and methods

### Setting

The data presented in this report were collected as a part of the Brigham and Women’s Wellness Initiative (B-Well), a prospective cohort study conducted from July 2014 to June 2015 among employees of Brigham and Women’s Hospital (BWH), an academic medical center in Boston, Massachusetts. Eligible employees could not be nurses or physicians, as there were separate programs for such employees. Employees expressed an interest in improving their physical activity and self- reported exercising fewer than 30 minutes per week.

At baseline, participants underwent a health assessment and were given a commercial accelerometer, the Fitbit Flex (Fitbit Inc, San Francisco, California), which has been shown to have moderate levels of validity for measuring steps and physical activity [16–18]. Participants were instructed to wear the Fitbit continuously during waking hours on their non-dominant wrist and charge it approximately twice weekly. The Fitbit Flex counts steps taken on a minute-by-minute basis and syncs via Bluetooth with the user’s computer or smartphone to send collected data to Fitbit Inc’s proprietary servers. Using the servers’ Application Program Interface (API), we downloaded minute-by-minute step counts for every subject in the study on a weekly basis.

Participants joined or were placed into three member teams at the onset of the study. Initially, participants spent two weeks in an introductory period where they earned monetary awards for wearing the Fitbit. During each successive week for 24 weeks, each participant could earn monetary rewards each week for either increasing the amount of time spent doing physical activity by at least 10% from the preceding week or by meeting CDC guidelines for moderate aerobic physical activity. Specifically, participants were given $10 for achieving their weekly physical activity goal and $15, $25, or $50 for achieving their goal for 4 weeks, 12 weeks, or 24 weeks respectively. All awards were doubled if all three members of a team reached their individual goal.

### Physical activity

During the course of the intervention, we determined valid days of data as those with at least 10 hours of activity recorded on the accelerometer [19]. Each valid day of data was analyzed for bouts of physical activity, which we defined as a period of at least ten minutes in which the wearer participated in moderate-to-vigorous physical activity, in accordance with CDC guidelines [11]. We set ranges of 100–174 steps/minute for moderate physical activity and ≥175 steps/minute for vigorous physical activity; a minute of vigorous physical activity counted as two minutes of moderate physical activity. The former range was selected to correspond with defined metabolic equivalents of task (METs) for moderate (≥3 METs) physical activity [20]; and given the limited study on the association between step cadence and vigorous (≥6 METs) physical activity, the 175 steps threshold was selected to be conservative.

We allowed for up to two grace minutes during which the participant could fall below the lower threshold; if the subject sustained activity at less than the threshold for more than two minutes, the bout was terminated. All bouts within a week were summed to determine the individual’s weekly physical activity. Days with fewer than 10 hours of activity recorded on the accelerometer were assumed to have no physical activity.

We calculated the mean of the weekly time spent in physical activity captured by the Fitbit for each participant over the course of the intervention (among weeks with at least 1 day of accelerometer wear) and stratified the participants into three physical activity groups: 0–74 min/week, 75–149 min/week, and ≥150 min/week. The choices of cut-off points were informed by the distribution of physical activity among the cohort (the median amount of weekly physical activity was 74 min/week) as well as CDC guidelines (at least 150 min/week).

### Absenteeism

At baseline, participants reported how many hours they were usually scheduled to work. Each week participants completed an online questionnaire reporting whether they were absent from work the previous week during times they would have been scheduled to work. They reported whether the absences were planned or unplanned, the reason for the absence (e.g. vacation, doctor’s appointment, illness), and the number of hours they were absent. This information was not disclosed to anyone outside the study staff team. Participants were entered into a raffle for a $25 gift card for each absenteeism questionnaire that they completed, with 1 in 250 questionnaires being selected to win.

At BWH, all full-time employees accrue 4.5 hours per week of paid time off annually, which includes sick time, discretionary leave, and scheduled holidays. Part-time employees who work more than 20 hours/week accrue paid time off proportionally to the number of hours worked.

### Analysis

We first compared baseline characteristics of participants by stratifying them into two groups defined by the presence or absence of any unplanned illness-related absence from work during the intervention period. Evaluated baseline characteristics included self-reported demographic data collected at baseline (including age, sex, education, race/ethnicity, and annual household income) and clinical data (including measured body mass index (BMI), self-reported number of daily medications taken, and self-reported chronic medical conditions). Educational attainment was categorized in two groups: high school or less and some college or higher. Body mass index (BMI) was categorized into four groups: BMI<25.0 (normal weight), BMI 25.0–29.9 (overweight), BMI 30.0–34.9 (class I obesity), and BMI ≥35.0 (class II–III obesity) [21, 22]. We asked study participants if they had chronic medical conditions (heart disease, hypertension, lung disease, diabetes, ulcer or stomach disease, kidney disease, liver disease, anemia or other blood disease, cancer, depression, arthritis, back pain, or any other self-reported medical problem) [23] and categorized them as having none, at least one, or not responding to the question.

The primary outcome of this analysis was the number of hours of unplanned absence from work due to illness during the 24 invention weeks. We used Poisson regression to determine factors associated with the greater rates of unplanned absence. Since each participant was scheduled for different levels of time at work and completed varying numbers of absenteeism questionnaires, individual person-time was defined as the number of hours scheduled to work during the weeks that participants completed that week’s absenteeism questionnaire; we assumed that an individual’s completed attendance questionnaires were representative of weeks where they did not report absenteeism. The primary independent variable of interest was physical activity (in three groups); we defined the effect of physical activity on unplanned absenteeism as the ratio of the rate of absenteeism in the two lower physical activity groups as compared to that in the highest physical activity group. We initially built bivariate models; covariates that reached a significance level of α = 0.15 and suggested a trend in the rate ratio were advanced into multivariable models. In addition, to examine whether physical activity can predict unplanned absenteeism, we built a model predicting the unplanned absenteeism occurring in the second part of the intervention, weeks 15–26 as a function of sufficient physical activity in the first 12 weeks of intervention, adjusting for age, sex, race, obesity, and comorbidity. Sufficient physical activity was defined as engaging in PA consistent with CDC PA guidelines for at least eight out of 12 weeks.

### Sponsorship, ethics statement, and data availability

The B-Well program and this present analysis were sponsored by the BWH Presidential Fund. The funder had no role in study design, data collection and analysis, decision to publish, or preparation of the manuscript. B-Well was approved by the hospital institutional review board, the Partners Human Research Committee, under protocol 2014P000970/BWH prior to its initiation and all participants provided written acknowledgement of informed consent prior to starting in the study. The study dataset is available to interested parties and is included as a supporting information file (S1 File).

## Results

### Cohort characteristics

Seven hundred seventy-seven individuals completed an eligibility survey, of whom 398 were eligible for the B-Well study. Three hundred persons enrolled and the remaining 98 were placed on a waitlist in the case that space became available prior to closure of enrollment. Eight participants did not wear their accelerometer for any amount of time, did not provide employment status in the baseline questionnaire or did not complete at least one absenteeism questionnaire and were excluded from this analysis, leaving 292 participants as the final analytical cohort (Table 1). The mean age was 38 years (SD 11), 243 (83%) were female and 193 (66%) were white. Nearly all (95%) had at least some college education and 40% reported an annual household income of more than $100,000. Thirty-eight percent were obese, one-quarter of participants reported taking at least two medications daily, and half reported having at least one chronic medical condition.

**Table 1:** Characteristics of study cohort, stratified by whether they were absent from work due to illness at any point during the intervention.

	Overall	No illness-related absences	Any illness-related absences	P-value
Sample Size	292	167	125	
Age [Mean (SD)]	38.5 (11.2)	36.8 (10.7)	40.7 (11.5)	0.0028
Sex [N (%)]				0.1153
Female	243 (83%)	134 (80%)	109 (87%)	
Male	49 (17%)	33 (20%)	16 (13%)	
Race [N (%)]				0.0191
White	193 (66%)	101 (60%)	92 (74%)	
Non-white	99 (34%)	66 (40%)	33 (26%)	
Education [N (%)]				0.9969
High school or less	14 (5%)	8 (5%)	6 (5%)	
Some college or higher	275 (95%)	157 (94%)	118 (94%)	
Missing	3 (1%)	2 (1%)	1 (1%)	
Household Income, annual [N (%)]				0.9458
$0 –$59,000	89 (31%)	49 (29%)	40 (32%)	
$60,000 –$99,000	84 (29%)	48 (29%)	36 (29%)	
More than $100,000	116 (40%)	68 (41%)	48 (38%)	
Not Disclosed	3 (1%)	2 (1%)	1 (1%)	
Chronic medical conditions [N (%)]				0.0267
0	116 (40%)	76 (46%)	40 (32%)	
1 or more	151 (52%)	75 (45%)	76 (61%)	
Did not report	25 (9%)	16 (10%)	9 (7%)	
Medications, daily number [N (%)]				0.1150
0	127 (44%)	80 (48%)	47 (38%)	
1	89 (31%)	52 (31%)	37 (30%)	
2–3	61 (21%)	29 (17%)	32 (26%)	
4–8	15 (5%)	6 (4%)	9 (7%)	
Body Mass Index (BMI) [N (%)]				0.0054
<25.0	92 (32%)	63 (38%)	29 (23%)	
25.0–29.9	88 (30%)	48 (29%)	40 (32%)	
30.0–34.9	56 (19%)	34 (20%)	22 (18%)	
≥35.0	56 (19%)	22 (13%)	34 (27%)	
Blood Pressure [Mean (SD)]				
*Systolic*	118.6 (14.5)	117.0 (13.0)	120.9 (16.0)	0.0214
*Diastolic*	74.3 (9.7)	73.5 (9.6)	75.3 (9.9)	0.1248
Weekly Physical Activity over the Intervention (mean minutes) [N (%)]				0.0128
0–74	148 (51%)	79 (47%)	69 (55%)	
75–149	83 (28%)	43 (26%)	40 (32%)	
150 or more	61 (21%)	45 (27%)	16 (13%)	

### Physical activity

Two-hundred ninety two participants contributed 38808 valid days (out of 49056; 79%) and 5465 valid weeks (out of 7008; 78%). On average, study participants contributed 19 out of 24 (79%) valid weeks. Over the course of the 24 weeks of intervention, participants wore their accelerometer for a mean of 21.5 weeks (median 24 weeks). Wear time steadily decreased over the course of the intervention, going from 96% of participants wearing their accelerometers during the first intervention week to 59% by the final intervention week. Participants walked an average of 8,500 steps per day (SD 2,700) and engaged in a mean of 90 minutes (SD 74) of physical activity weekly; the Pearson correlation between total steps and total physical activity was 0.71.

Over the course of the intervention, 148 (51%) participants exercised a mean of 74 or fewer minutes weekly, 83 (28%) exercised 75–149 minutes on average weekly, and 61 (21%) averaged at least 150 minutes weekly. Overall, 110 participants (38%) did not meet CDC guidelines of 150 minutes of physical activity in any of the 24 intervention weeks, while 118 participants (40%) met the guidelines between 1 and 12 times and 64 participants (22%) met the guidelines between 13 and 24 times.

### Absenteeism

Eighty-four percent of possible weekly attendance questionnaires were completed, with 77% of study participants completing at least 20 absenteeism questionnaires over the 24 weeks of the intervention. Enrolled participants reported that they would have been scheduled to work 991 hours (SD 177) over the course of the intervention (slightly more than 40 hours per week). Participants were absent from work for any reason (including illness, holidays, planned vacation, doctor’s appointments and other reasons) a mean of 86 hours (SD 83) over the course of the study. On average, participants reported missing 24 hours (SD 42) of work due to unplanned reasons, with half of the unplanned hours (mean 14, SD 38 hours) due to illness.

### Association between physical activity and absenteeism

Based on the available data, study participants with physical activity levels meeting CDC guidelines (≥150 min/week) on average missed 5 hours of work during the intervention period due to illness compared to 11 hours among participants in the middle physical activity group (75–149 min/week) and 19 hours among participants in the lowest physical activity group (0–74 min/week). In the unadjusted model, compared to the highest physical activity group (reaching CDC guidelines), those in the medium physical activity group had a 2.7 (95% CI 1.4–5.2) fold higher rate of unplanned absenteeism due to illness and those in the lowest physical activity group had a 4.1 (95% CI 2.0–8.4) fold higher rate of illness-related absenteeism compared to those in the highest physical activity groups.

**Fig 1:**
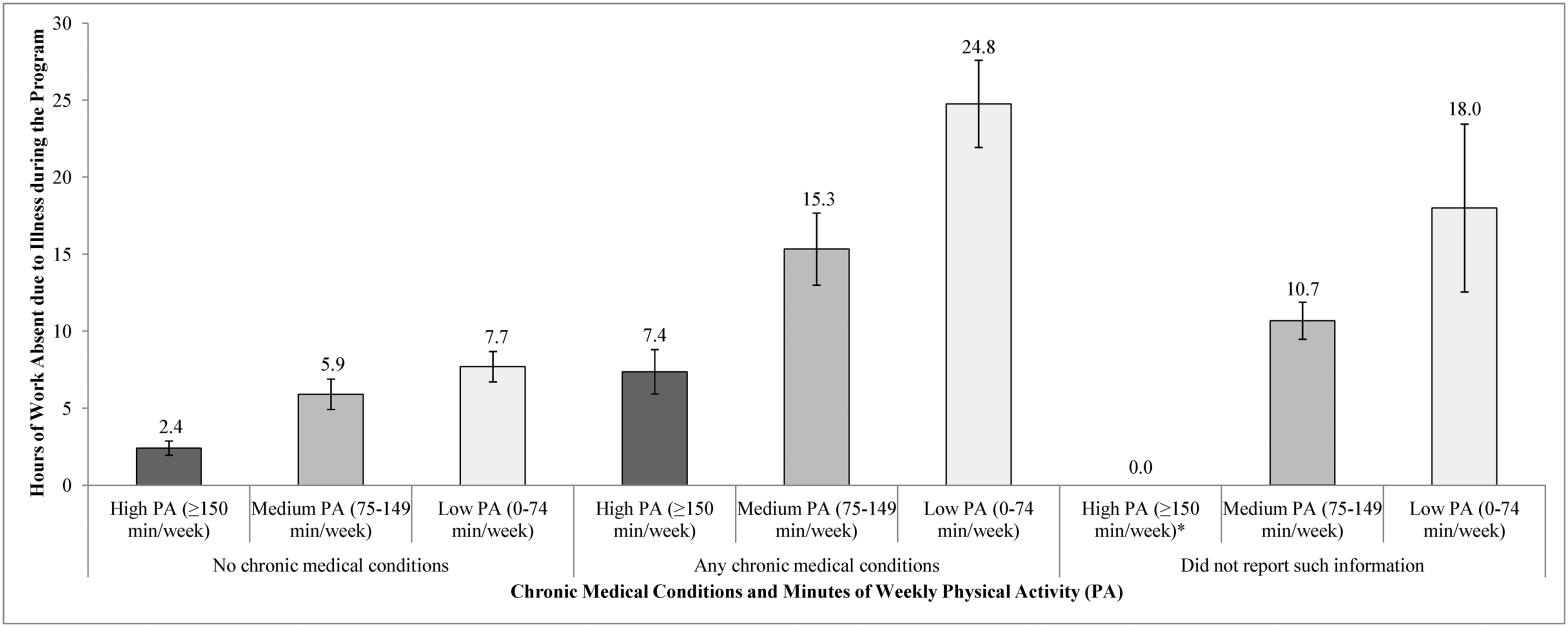
Unplanned hours absent from work due to illness, by presence of chronic medical conditions and amount of weekly physical activity. The chart describes relationship between the unadjusted amount of time absent from work due to illness over the course of the intervention, stratified by amount of weekly physical activity (≥150 minutes/week, 75–149 minutes/week, and 0–74 minutes/week) and presence of chronic medical conditions (none, at least one, or not reporting), along with associated 95% confidence intervals. Note that no individuals both achieved ≥150 minutes/week of physical activity and did not report comorbidity information.

In each physical activity group the presence of chronic medical conditions influenced the rate of unplanned absenteeism due to illness (Fig 1). Among those meeting CDC guidelines, the unplanned absenteeism due to illness was 2 hours among those without chronic medical conditions compared to 8 hours among those with at least one chronic medical condition. In the medium physical activity group the rates of unplanned absenteeism was 6 hours for those without chronic medical conditions compared to 15 hours to those with at least one chronic medical condition. Among those in lowest physical activity group, study participants with no chronic medical conditions missed 8 hours due to illness compared to 25 hours among those with at least one chronic medical condition. After adjusting for age, race, education and number of chronic medical conditions, compared to the highest physical activity group those in the middle physical activity group had a 2.4 (95% CI: 1.3–4.5) fold higher rate and those in the lowest physical activity group had a 3.5 (95% CI: 1.7–7.2) fold higher rate of unplanned absenteeism due to illness (Table 2).

**Table 2:** Association between the rate of unplanned absenteeism due to illness and demographic, clinical factors and physical activity.

	Bivariate Analysis			Multivariable Analysis		
	Rate Ratio*	95% CI	p-value	Rate Ratio*	95% CI	p-value
Age (in 5 year increments)	1.230	1.114–1.358	<0.0001	1.169	1.061–1.287	0.0241
Sex			0.7620			
Female	(reference)					
Male	1.228	0.365–4.126				
Race			0.0113			0.0321
White	2.227	1.333–3.720		1.664	1.089–2.543	
Non-white	(reference)			(reference)		
Education			0.1347			0.1255
High school or less	(reference)			(reference)		
Some college or higher	1.845	0.836–4.071		2.873	0.960–8.595	
Household income, annual			0.2010			
$0 –$59,000	(reference)					
$60,000 –$99,000	1.519	0.654–3.529				
More than $100,000	1.661	0.981–2.813				
Not Disclosed	0.288	0.056–1.470				
Chronic medical conditions, number			0.0026			0.0102
0	(reference)			(reference)		
1 or more	3.294	1.924–5.639		2.357	1.477–3.761	
Did not report	2.830	1.195–6.703		2.073	0.814–5.275	
Medications, daily number			0.7988			
0	(reference)					
1	0.887	0.409–1.926				
2–3	1.170	0.524–2.615				
4–8	1.228	0.466–3.233				
Body mass index (BMI)			0.0643			
Normal: <25.0	(reference)					
Overweight: 25.0–29.9	2.607	1.129–6.020				
Obese: 30.0–34.9	1.448	0.706–2.971				
Morbidly Obese: ≥35.0	2.335	1.231–4.429				
Weekly physical activity, mean minutes			0.0011			0.0068
0–74	4.106	2.007–8.402		3.495	1.688–7.238	
75–149	2.720	1.423–5.199		2.387	1.260–4.521	
150 or more	(reference)			(reference)		

* Rate ratios were derived from the Poisson regression model built to determine factors associated with the greater rate of unplanned absence per person-time, where person-time was defined as the number of hours scheduled to work during the weeks where participants completed the absenteeism questionnaire.

### Other factors related to unplanned absenteeism due to illness

Results of multivariable analyses showed that white study participants had a 1.7 (95% CI: 1.1–2.5) fold greater the rate of unplanned absenteeism due to illness compared to non-white study participants. Those with chronic medical conditions had twice the rate of unplanned absenteeism due to illness compared to the study participants without chronic medical conditions. Every five-year increase of age was associated with 1.2 fold greater rate of unplanned absenteeism due to illness (Table 2). We further evaluated the effect of BMI on work absence in the multivariable analysis mentioned above but did not see a significant effect of BMI on rate of unplanned absenteeism.

Sufficient physical activity during the first 12 weeks of the intervention was associated with fewer hours of unplanned absenteeism during the second half of the intervention (2.9 vs. 7.5 hours of unplanned absenteeism due to illness for those who complied with CDC PA guidelines for at 8 out of 12 weeks vs. those who did not). After adjusting for age, sex, race, education, comorbidities and BMI the rate of unplanned absenteeism due to illness among those who were not sufficiently physically active during the first 12 weeks of intervention was 2.87 (95% CI: 1.16, 7.09) higher compared to those who were sufficiently active during the first half of the intervention.

## Discussion

In this study evaluating a cohort of employees undergoing a physical activity intervention, we quantified the relationship between objectively-measured physical activity and unplanned illness-related absenteeism. Our findings suggest that less physically active individuals, as well as persons with any number of chronic medical issues, had higher rates of unplanned illness-related absenteeism.

These findings are consistent with previous work regarding long-term disability leave which have found that persons who engage in less physical activity are more likely to be absent from work [24, 25]. Increasing physical activity has been shown to reduce the future risk of both short-term absenteeism and long-term disability leave [26–28]. Patel et al. have recently demonstrated in a randomized controlled trial that there are a variety of feasibly implementable methods that are efficacious at increasing physical activity in the workplace [29, 30]. Workplace wellness programs that broadly involve individual counseling, education, and on-site group activities have generally been shown to have a positive return on investment by reducing health care costs as well as overall absenteeism [8]. However, some groups have indicated difficulty in ensuring that their program translates short-term benefits into long-term changes in their physical activity [31–35]. Many workplace health promotion programs were not able to capture the full scope of an individual’s activities outside of the workplace; with commercial wearable devices now widely available, this limitation has lessened. Participants in such programs now can objectively measure physical activity over the long-term.

In our cohort, we observed that non-whites and persons with less education are less likely to be absent from work due to illness, in contrast to previous studies that have found that socioeconomic status is negatively linked to both general health and illness-related absenteeism [36–39]. This may be because the level of household income and education were generally high in our cohort, and paid leave was both universally available and standardized. Study participants likely had differing views on how severe illness must be before one takes time off and potentially may have had concerns regarding use of sick leave and their job security. Furthermore, our analyses highlight the independent effect of having chronic medical conditions on absenteeism at every level of physical activity. A large body of literature suggests that consistent physical activity leads to lower rates of chronic medical conditions [40, 41]. We hypothesize from our data that physical activity interventions among younger adults may have a stronger effect on unplanned absenteeism due to illness than would be predicted by increases in physical activity alone.

The results of our analysis should be viewed considering several limitations. Our analysis took place in the context of an uncontrolled health promotion initiative designed to improve physical activity using financial incentives among health-conscious, highly educated, predominantly white female sedentary volunteers. Relationships discussed in this analysis should be viewed as associations and not causal pathways. During screening, we did not systematically record the reason that subjects were considered ineligible. Additionally, not all persons completed absenteeism questionnaires on every occasion. We attempted to maximize completion rates by assuring study participants that their responses would not be communicated to any supervisor, offering a weekly lottery for those who completed the survey, and administering the questionnaire electronically. We captured data on absenteeism for over 80% of the potential participant-weeks and assumed that incomplete weeks were similar to weeks where data was reported. While there appeared to be a trend toward lower completion rate for those study participants who engaged in lower amounts of physical activity, we accounted for the differential completion rate in our analyses. While previous absenteeism due to illness would have been a strong predictor of absence during our intervention, we did not collect information on pre-intervention absence. Additionally, to ensure that study participants would not be overburdened by the number of questions asked at each time point, we collected the data on chronic medical conditions only during the three-month follow-up questionnaire and do not have an accurate assessment of the number of chronic medical conditions on all study participants. We found that those participants who did not return three-month questionnaire had a similar rate of unplanned absenteeism and were less likely to be physically active as compared to persons with chronic medical conditions. Finally, the activity monitor we used (Fitbit Flex) captures only ambulatory physical activity and not other forms of aerobic activity (such as cycling or swimming). Participants wore their Fitbit for an average of 21.5 weeks over the 24-week intervention. We excluded participants’ data on days where the activity monitor recorded any number of steps for fewer than 10 hours. While we may not have fully captured all physical activity through the Fitbit, because study participants were compensated for only objectively measured physical activity, we believe that there was unlikely to have been substantial amounts of physical activity during non-wear time.

In summary, we present data from a pragmatic workplace-based intervention that objectively measured physical activity among employees who were provided with financial incentives. Our data on the influence of physical activity on short-term illness-related absenteeism lead to several important implications. Noting the substantial association between lower physical activity levels and unanticipated illness, employers and policymakers can consider the value of workforce-based interventions to increase physical activity. However, it remains to be assessed whether programs can be designed to translate increases in physical activity into improved work attendance rates [42–44]. Such programs may offer a promising opportunity to reduce unplanned absenteeism from work, and the efficacy, cost, and benefit of such programs should thus be further evaluated.

## Supporting information

S1 File Analytic dataset. (XLS)
